# Computational Identification of Guillain-Barré Syndrome-Related Genes by an mRNA Gene Expression Profile and a Protein–Protein Interaction Network

**DOI:** 10.3389/fnmol.2022.850209

**Published:** 2022-03-17

**Authors:** Chunyang Wang, Shiwei Liao, Yiyi Wang, Xiaowei Hu, Jing Xu

**Affiliations:** ^1^Department of Neurology, Tianjin Medical University General Hospital, Tianjin, China; ^2^Tianjin Key Laboratory of Cerebral Vascular and Neurodegenerative Diseases, Department of Neurorehabilitation and Neurology, Tianjin Huanhu Hospital, Tianjin Neurosurgical Institute, Tianjin, China; ^3^Department of Neurology, Tianjin Haihe Hospital, Tianjin, China

**Keywords:** GBS, genes, mRNA microarray analysis, protein–protein interaction, shortest path

## Abstract

**Background:**

In the present study, we used a computational method to identify Guillain–Barré syndrome (GBS) related genes based on (i) a gene expression profile, and (ii) the shortest path analysis in a protein–protein interaction (PPI) network.

**Materials and Methods:**

mRNA Microarray analyses were performed on the peripheral blood mononuclear cells (PBMCs) of four GBS patients and four age- and gender-matched healthy controls.

**Results:**

Totally 30 GBS-related genes were screened out, in which 20 were retrieved from PPI analysis of upregulated expressed genes and 23 were from downregulated expressed genes (13 overlap genes). Gene ontology (GO) enrichment and KEGG enrichment analysis were performed, respectively. Results showed that there were some overlap GO terms and KEGG pathway terms in both upregulated and downregulated analysis, including positive regulation of macromolecule metabolic process, intracellular signaling cascade, cell surface receptor linked signal transduction, intracellular non-membrane-bounded organelle, non-membrane-bounded organelle, plasma membrane, ErbB signaling pathway, focal adhesion, neurotrophin signaling pathway and Wnt signaling pathway, which indicated these terms may play a critical role during GBS process.

**Discussion:**

These results provided basic information about the genetic and molecular pathogenesis of GBS disease, which may improve the development of effective genetic strategies for GBS treatment in the future.

## Background

As a result of damage to the peripheral nervous system, Guillain–Barré syndrome (GBS) is characterized by rapid-onset muscle weakness. The exact molecular mechanism and epigenetic feature of this disease are still unclear. Therefore, it is of great importance to identify GBS-related genes that could be used as a biomarker for early diagnosis and effective genetic strategies for clinical therapies.

In biomedicine and genomics, trying to identify the disease genes has become one of the most critical and challenging problems. Gene expression profiles can be used to select differentially expressed genes as disease genes. These methods are useful resources and have been widely used (Cai et al., 2010; Huang et al., 2010, 2011; Liu et al., 2010). However, it has not been well solved about the errors and false-positive problem in the high-throughput data (Li et al., 2012b). Thus, it is not a good idea to use only the gene expression profiles to identify novel genes.

According to the “guilt by association” rule, interacting proteins share the same or similar functions, which may participate in the same pathway. First proposed by Nabieva et al. (2005) disease-related genes could be identified from protein–protein interaction (PPI) networks based on existing PPI data. Methods based on the PPI data have been widely used for gene function predictions.

In the present study, we used a computational method to search GBS-related genes through integrating a gene expression profile and a weighted functional PPI network, which may improve the defect of using high-throughput data only. Previous studies also have successfully applied this integrating method for identifying novel genes in various diseases, for example, the influenza A/H7N9 virus infection (Zhang et al., 2014), colorectal cancer (Li et al., 2012b,2013a), lung cancer (Li et al., 2013b), HBV virus infection-related hepatocellular carcinoma (Jiang et al., 2013), retinoblastoma (Li et al., 2012c), etc.

## Materials and Methods

### Implementation

**FIGURE 1:**
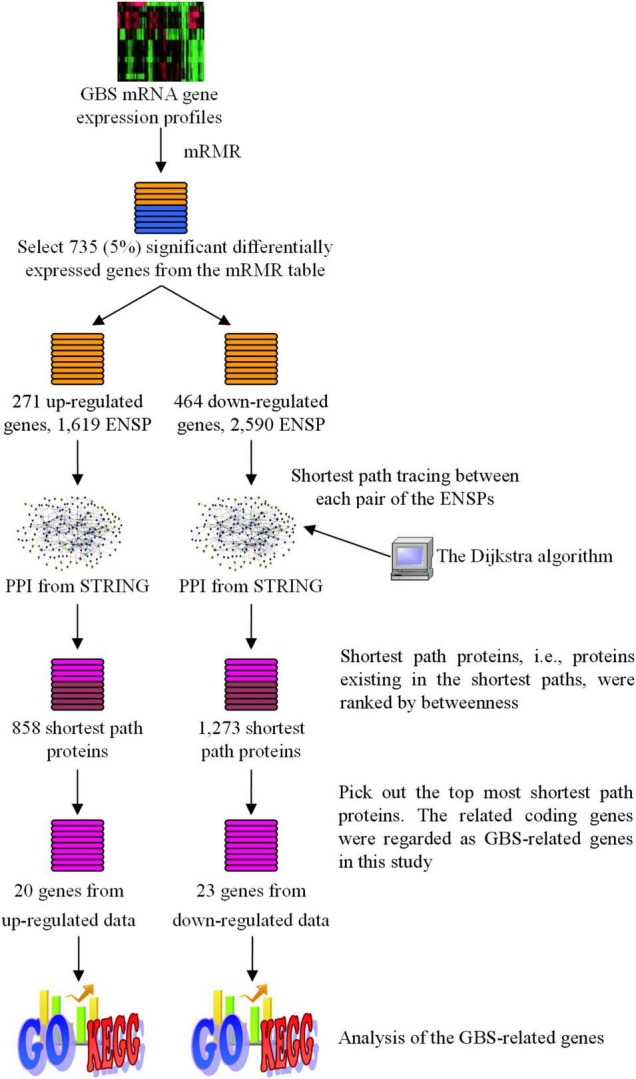
The flowchart of the method developed in this study to identify GBS-related human genes. Target Human Proteins interacted with GBS-related genes were obtained based on sharing GO terms. Shortest path proteins were calculated from the shortest paths between every pair of the Target Human Proteins, by searching by the Dijkstra algorithm in the network constructed from STRING. Finally, for shortest path proteins from the analysis of upregulated genes, top 20 proteins (20 genes) with betweenness > 1,400 were selected, while for downregulated genes the top 24 proteins (23 genes) with betweenness > 4,000 were selected.

4273π: Bioinformatics Figure 1 showed the total procedure of our method. In the following sub-sections, details are as follows:

### mRNA Expression Profiles of Guillain–Barré Syndrome Patients and Healthy Controls

mRNA Microarray analyses were performed on the peripheral blood mononuclear cells (PBMCs) of 4 GBS patients and 4 age- and gender-matched healthy controls. Baseline characteristics are shown in Table 1. Fulfilled the standard diagnostic criteria, GBS patients were recruited from Tianjin Medical University General Hospital (Asbury, 1981). Patients manifested as symmetrical flaccid weakness and decreased reflexes in the absence of alternative causes with cerebrospinal fluid albumincytological dissociation, and electrodiagnostic evidence of neuropathy (Shahrizaila et al., 2021). Blood samples were collected within the peak timing of manifesting GBS and before using glucocorticoid, intravenous immune globulin (IVIG), or plasma exchange. Before enrollment, informed consent was signed from all involved patients. This study was approved by the ethical review committees of Tianjin Medical University General Hospital. Human peripheral blood mononuclear cells (PBMCs) were isolated from all GBS patients and healthy controls. RNA extraction and production of labeled cRNA were conducted as our previous study (Xu et al., 2016). Standard data analyses are provided for RNA quality control. The labeled cRNAs were designed for the global profiling of human lncRNAs, mRNAs, and protein-coding transcripts by hybridizing onto the human LncRNA Expression Microarray V3.0 (Arraystar, Rockville, MD).

**TABLE 1:** Baseline characteristics of GBS patients and healthy controls.

	GBS patients	Healthy controls
**Age (years)**		
Median (IQR)	55 (49–59)	56 (46–60)
**Sex**		
Men	2 (50%)	2 (50%)
Women	2 (50%)	2 (50%)
**Days from onset to PBMC extraction**		
Mean (range)	5 (4–6)	–

Totally an expression profile dataset of 8 samples, 21,620 probes was obtained. Then signal intensity was log2 transformed and normalized and 14,707 genes were derived from source probes.

### The mRMR Method

The maximum relevance minimum redundancy (mRMR) method (Peng et al., 2005; Li et al., 2012a,b; Zhang et al., 2012) was employed to rank the importance of total 14,707 genes examined, according to the Maximum Relevance Minimum Redundancy criterion. Each gene was recognized as a feature during this procedure. Features most important in distinguishing GBS patients and healthy controls were selected according to the Maximum Relevance criterion. Meanwhile, features containing redundant information were excluded by the Minimum Redundancy criterion, according to the previously described procedure (Xu et al., 2016). In brief, two values were calculated using mRMR criteria: value A for relevance and value B for redundancy. The feature is measured using the value A–B. The features is correlated with value A–B (Peng et al., 2005; Li et al., 2012a,b; Zhang et al., 2012).

The mRMR method was used to generate two ordered list: the MaxRel Table and mRMR Table. All the features were ranked only by the Maximum Relevance criterion in the MaxRel Table, while they were ranked by the mRMR criteria in the mRMR Table. The two tables are provided in Supplementary Material 1.

### Protein–Protein Interaction Data From Search Tool for the Retrieval of Interacting Genes

As an online database resource, search tool for the retrieval of interacting genes (STRING) (Szklarczyk et al., 2011)^1^ compiles both experimental and predicted protein–protein interactions with a confidence score to quantify each interaction confidence. STRING retrieved a weighted PPI network. The proteins in that network are expressed as nodes. Edges marked with confidence scores marked interactions between proteins if they interacted with each other. Interacting proteins in the PPI network share much more similar biological functions than non-interactive proteins (Kourmpetis et al., 2010; Ng et al., 2010; Szklarczyk et al., 2011). The explanation is the protein and its interactive ones may from the same protein complex carrying a specific function or may participate in one pathway.

In this study, we used STRING (DAVID 11.5) to construct a graph G with the PPI data. Pathway analysis was performed using DAVID.^2^

In that graph, proteins were represented as nodes. A *d* value, not a confidence score (s) was assigned to the weight of each interaction edge. The *d* value was calculated according to the equation*d* = 1000×(1−*s*). Therefore, *d* value represented protein distances to each other: the smaller distance is correlated with a higher interaction confidence score and more similar functions.

In the present work, we analyzed every two protein interactions from the significant differentially expressed proteins.

### Shortest Path Tracing

We used the Dijkstra algorithm to find the shortest path in the graph *G* between two given proteins. In this study, the Dijkstra algorithm was implemented with R package “igraph.” A shortest path was traced from each 1,619 proteins to all the other ones in the graph, which was for the upregulated genes. For downregulated genes, the shortest path of each of the 2,590 proteins to all the other ones was traced in the graph.

Then we picked out all proteins existing on the shortest paths and ranked these proteins according to their betweenness. For upregulated genes, 858 shortest path proteins were retrieved, while for downregulated genes, 1,273 shortest path proteins were retrieved, as list in Table 2. The 858 and 1,273 shortest path proteins were sequenced using betweenness, respectively.

**TABLE 2:** Number of genes or proteins in each step of our computational procedures.

	Significant differentially expressed genes by mRMR		Shortest path proteins	Shortest path proteins with betweenness > threshold	Final GBS related genes
	Genes	Proteins(ENSP)			
Upregulated	271	1,619	858	20	20
Downregulated	464	2,590	1,273	24	23

The betweenness threshold should be set in order to select significant ones from a ranked list. By a computational method, we can set the threshold differently to yield a different number of gene results. The more the threshold is, the less the genes are. Generally speaking, it is practical to select the top most 20–30 significant genes for further analysis or for experimental validation. Furthermore, the threshold values should be different for upregulated genes and for downregulated ones, because the number of path tracing proteins and the number of shortest path proteins were different.

In this study, for shortest path proteins from the analysis of upregulated genes, top 20 proteins (20 genes) with betweenness > 1,400 were selected, while for downregulated genes the top 24 proteins (23 genes) with betweenness > 4,000 were selected. These 20+23 genes were regarded as the final significant GBS-related genes in the present work and they were list in Tables 3, 4, respectively.

**TABLE 3:** The 20 GBS-related genes identified by PPI network from the analysis of upregulated expressed genes from the expression profiles.

ENSP	Gene	Betweenness
ENSP00000269305	TP53	13,036
ENSP00000344818	UBC	6,065
ENSP00000344456	CTNNB1	4,569
ENSP00000275493	EGFR	3,294
ENSP00000263253	EP300	2,807
ENSP00000326366	PSEN1	2,456
ENSP00000417281	MDM2	2,445
ENSP00000270202	AKT1	2,350
ENSP00000221494	SF3A2	2,184
ENSP00000264657	STAT3	2,182
ENSP00000339007	GRB2	2,150
ENSP00000324806	GSK3B	2,094
ENSP00000284981	APP	2,046
ENSP00000357879	PSMD4	1,754
ENSP00000350941	SRC	1,655
ENSP00000356425	UCHL5	1,614
ENSP00000361626	YBX1	1,574
ENSP00000338018	HIF1A	1,444
ENSP00000262613	SLC9A3R1	1,438
ENSP00000252486	APOE	1,410

**TABLE 4:** The 23 GBS-related genes identified by PPI network from the analysis of downregulated expressed genes from the expression profiles.

ENSP	Gene	Betweenness
ENSP00000269305	TP53	36,055
ENSP00000344818	UBC	15,309
ENSP00000275493	EGFR	12,072
ENSP00000344456	CTNNB1	12,052
ENSP00000270202	AKT1	10,629
ENSP00000339007	GRB2	10,496
ENSP00000221494	SF3A2	9,484
ENSP00000206249	ESR1	8,165
ENSP00000263253	EP300	7,353
ENSP00000264657	STAT3	6,262
ENSP00000350941	SRC	6,111
ENSP00000417281	MDM2	6,002
ENSP00000362649	HDAC1	6,001
ENSP00000348461	RAC1	5,995
ENSP00000329357	SP1	5,560
ENSP00000361626	YBX1	5,343
ENSP00000264033	CBL	5,062
ENSP00000337825	LCK	4,852
ENSP00000314458	CDC42	4,798
ENSP00000304903	CD2BP2	4,549
ENSP00000358490	CD2	4,549
ENSP00000324806	GSK3B	4,281
ENSP00000046794	LCP2	4,043

## Results and Discussion

### Guillain–Barré Syndrome-Related Genes

In this study, we select the top 5%, i.e., 735 features, from the mRMR Table. These genes were considered to be significant differentially expressed genes according to the expression profiles and were analyzed in further procedures. In the 735 significant genes, there were 271 upregulated genes and 464 downregulated genes, producing 1,619 and 2,590 protein products, respectively. The upregulated genes and downregulated genes were analyzed, respectively, in the next procedures. The number of the genes and proteins is summarized in Table 2.

As shown in Tables 3, 4, 20 GBS-related genes were identified from the analysis of upregulated significant expressed genes from the gene expression profile data, while 23 GBS-related genes were identified from the data of downregulated ones.

From Tables 3, 4, it can be seen that there were 13 overlap genes, which were TP53, UBC, CTNNB1, EGFR, EP300, MDM2, AKT1, SF3A2, STAT3, GRB2, GSK3B, SRC, and YBX1. These genes were identified both from upregulated analysis and downregulated analysis, indicating they could play more important roles in GBS.

As an important tumor suppressor gene, TP53 takes part in cellular senescence, apoptosis and cell cycle progression (McCubrey et al., 2016). MDM2 has p53-independent transcription factor-like effects in nuclear factor-kappa beta (NF-κB) activation. Therefore, MDM2 promotes tissue inflammation and MDM2 inhibition has potent anti-inflammatory effects in tissue injury (Ebrahim et al., 2015). Increased levels of STAT3 proteins were observed in CD4+ T cells from GBS patients (Liu et al., 2015). Previous study showed that Grb2 promotes the correlation of FasL with adaptin beta. Moreover, in Schwann cells, Grb2 also helps FasL sorting to the cell surface. FasL potentially regulated cell death. Therefore, its cell surface localization is important for controlling local tissue remodeling and inflammation (Thornhill et al., 2008).

In recent years, high-throughput studies and candidate gene studies verified differential expression genes in GBS patients (Safa et al., 2021). In our previous study, we found 114 differentially expressed lncRNAs and 310 differentially expressed mRNAs between GBS patients and healthy controls, in which several gene ontology (GO) terms, such as cytosol, cellular macromolecular complex assembly, cell cycle, ligase activity, protein catabolic process were enriched in gene lists (Xu et al., 2016).

### Protein–Protein Interaction Relationship Between the Guillain–Barré Syndrome-Related Genes

**FIGURE 2:**
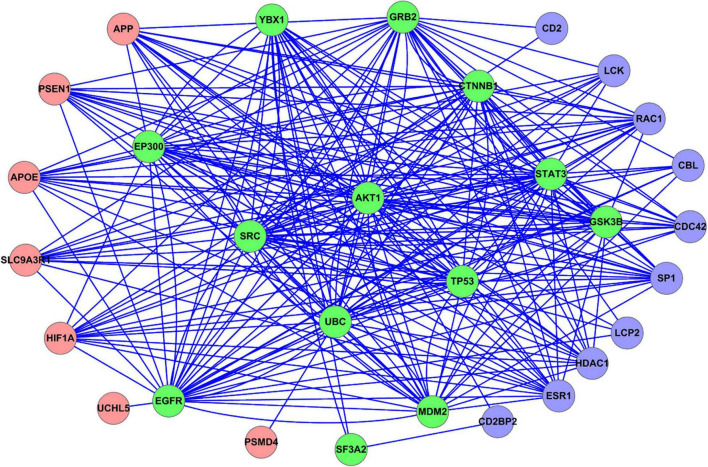
PPI relationship between all the GBS-related genes identified in this study. Red circle represents the GBS-related genes identified from upregulated analysis. Blue circle represents the GBS-related genes identified from downregulated analysis. Green circle represents the overlap GBS-related genes both from upregulated and downregulated analysis.

We mapped all the GBS-related genes to the PPI network constructed from the STRING database. The PPI relationships between the GBS-related genes were shown in Figure 2. The coding genes of the proteins were denoted as nodes. The 20 GBS-related genes identified from upregulated analysis were represented as red circles, while the 23 genes from downregulated ones were represented as blue circles. Note that there were 13 overlap genes, which were represented as green circles.

**FIGURE 3:**
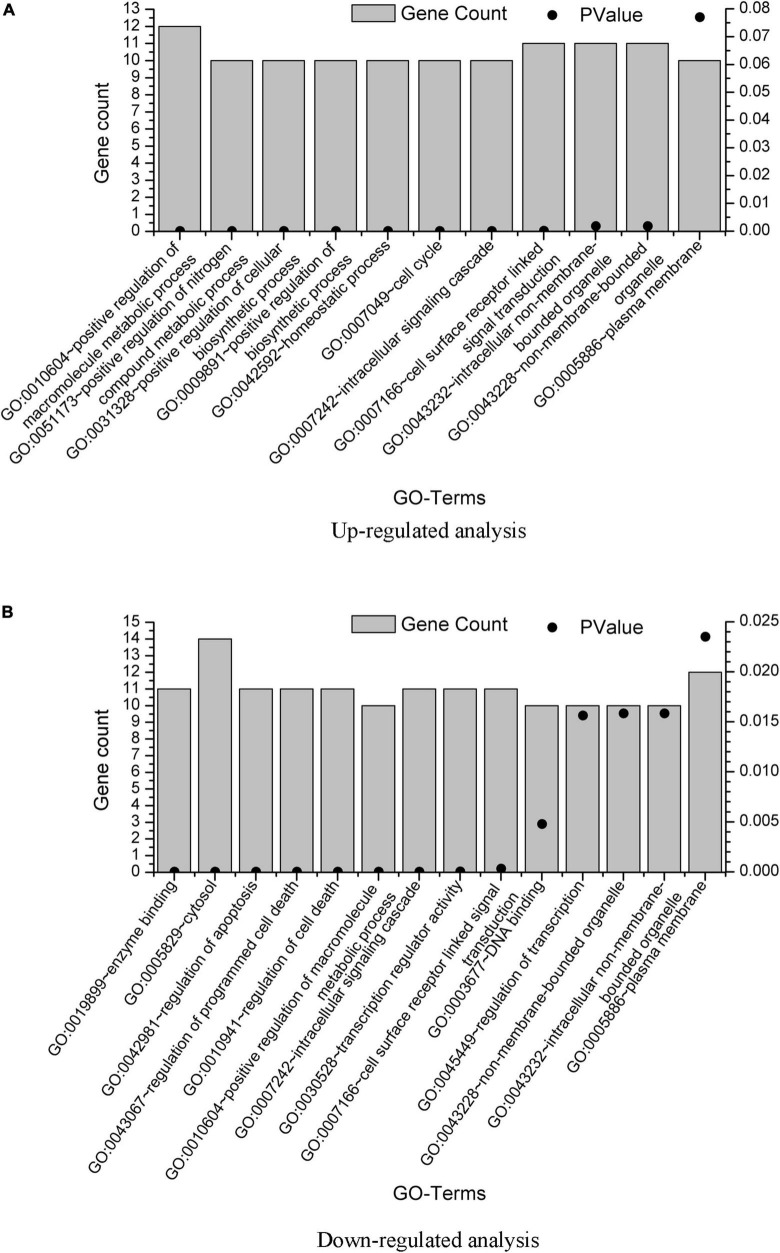
GO term enrichment analysis of the GBS-related genes. GO term enrichment analysis was performed on the 20 genes from upregulated analysis and 23 genes from downregulated analysis, with results plotted in **(A,B)**, respectively. Only pathways with gene count = 10 were shown.

The DAVID results are provided in Supplementary Material 2. We also plot the GO enrichment results in Figure 3 from the data in Supplementary Material 2. The overlap GO terms in both upregulated and downregulated analysis were listed as follows:

GO:0010604∼positive regulation of macromolecule metabolic process
GO:0007242∼intracellular signaling cascade
GO:0007166∼cell surface receptor linked signal transduction
GO:0043232∼intracellular non-membrane-bounded organelle
GO:0043228∼non-membrane-bounded organelle
GO:0005886∼plasma membrane

### KEGG Pathway Enrichment Analysis

**FIGURE 4:**
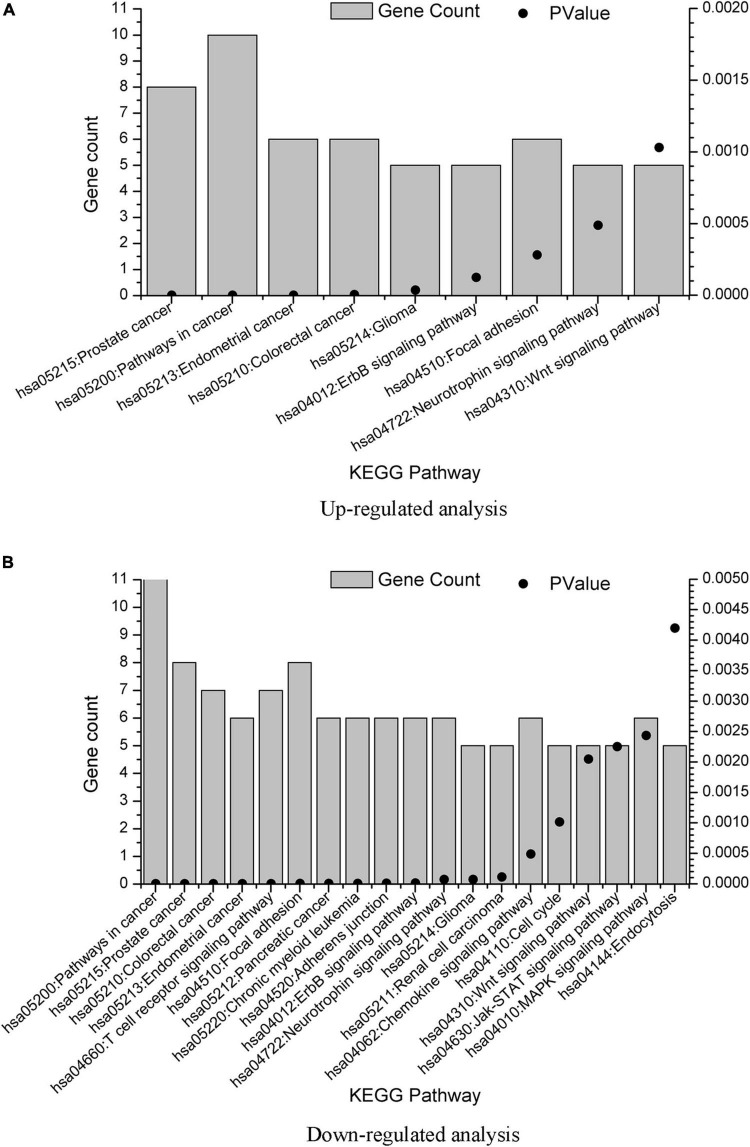
KEGG term enrichment analysis of the GBS-related genes. KEGG term enrichment analysis was performed on the 20 genes from upregulated analysis and 23 genes from downregulated analysis, with results plotted in **(A,B)**, respectively. Only pathways with gene count = 5 were shown.

Associated signaling pathways were analyzed using DAVID. Using Benjamin multiple testing correction method, the enrichment *p*-value was corrected to control family-wide false discovery rate under a certain rate (e.g., ≤ 0.05). The results were provided in Supplementary Material 3. We also plot the KEGG pathway enrichment results in Figure 4 from the data in Supplementary Material 3. The overlap KEGG pathway terms in both upregulated and downregulated analysis were listed as follows:

hsa05215: Prostate cancer
hsa05200: Pathways in cancer
hsa05213: Endometrial cancer
hsa05210: Colorectal cancer
hsa05214: Glioma
hsa04012: ErbB signaling pathway
hsa04510: Focal adhesion
hsa04722: Neurotrophin signaling pathway
hsa04310: Wnt signaling pathway

As one of the promising biomolecules, neurotrophins involved in the modulation of synaptic activity, neuronal survival, and release of neurotransmitters. There were released naturally post-injury with the potential to exhibit better functional recovery (Pandey and Mudgal, 2021). In the present study, we found Neurotrophin signaling pathway in both upregulated and downregulated pathway, which also indicates the role of neurotrophin in GBS pathological mechanism and nerve recovery.

Previous study found that Wnt/β-catenin signals participated in Schwann cell proliferation and apoptosis and acted as positive regulators of myelination (Lush and Piotrowski, 2014). Liu et al. (2020) also demonstrated that Wnt/β-catenin signaling pathway was upregulated in experimental autoimmune neuritis (EAN) rats. In the present study, Wnt signaling pathway was found in both upregulated and downregulated analysis, which was consistent with the above studies.

## Conclusion

In biomedicine and genomics, how to identify disease genes is one of the most critical and challenging problems. In this study, using the PPI network, we developed a computational method to search GBS-related genes based on the shortest paths. Totally, 30 most significant genes were screened out, which may imply their direct or indirect effects on the development of GBS, providing clues for further research and experimental validations. These findings may give a new reference for research into GBS pathogenesis and for new strategies for clinical therapies.

## Data Availability Statement

The original contributions presented in the study are included in the article/Supplementary Material, further inquiries can be directed to the corresponding author/s.

## Ethics Statement

The study was approved by the Ethics Committee of Tianjin Medical University General Hospital. The patients/participants provided their written informed consent to participate in this study.

## Author Contributions

CW, SL, and YW contributed to data curation and formal analysis. JX contributed to project administration, funding acquisition, and supervision. XH collected the data. All authors read and approved the final manuscript.

## Conflict of Interest

The authors declare that the research was conducted in the absence of any commercial or financial relationships that could be construed as a potential conflict of interest.

## Publisher’s Note

All claims expressed in this article are solely those of the authors and do not necessarily represent those of their affiliated organizations, or those of the publisher, the editors and the reviewers. Any product that may be evaluated in this article, or claim that may be made by its manufacturer, is not guaranteed or endorsed by the publisher.

## Footnotes

1
http://string.embl.de/

2
https://david.ncifcrf.gov/

## Supplementary Material

Supplementary Material 1 The MaxRel Table and the mRMR Table generated by the mRMR method. To evaluate the importance of all the genes in discriminating GBS and healthy controls from expression profiles, each gene was regarded as a feature and was ranked by only the Maximum Relevance criterion (the MaxRel Table), and by the Maximum Relevance Minimum Redundancy criterion (the mRMR Table). The smaller the index was in the mRMR Table, the more important the gene was.

Supplementary Material 2 Results of the GO term enrichment analysis on the GBS-related genes by the functional annotation tool DAVID. We performed the analysis on the 20 GBS-related genes from upregulated analysis and on the 23 GBS-related genes from downregulated analysis, respectively. Results only contained the GO terms having genes more than 10.

Supplementary Material 3 Results of the KEGG pathway enrichment analysis on the GBS-related genes by the functional annotation tool DAVID. We performed the analysis on the 20 GBS-related genes from upregulated analysis and on the 23 GBS-related genes from downregulated analysis, respectively. Results only contained the KEGG pathways having genes more than 5.
